# Study of Camelpox Virus Pathogenesis in Athymic Nude Mice

**DOI:** 10.1371/journal.pone.0021561

**Published:** 2011-06-28

**Authors:** Sophie Duraffour, Patrick Matthys, Joost J. van den Oord, Tim De Schutter, Tania Mitera, Robert Snoeck, Graciela Andrei

**Affiliations:** 1 Rega Institute, Laboratory of Virology and Chemotherapy, K.U.L, Leuven, Belgium; 2 Rega Institute, Laboratory of Immunobiology, K.U.L, Leuven, Belgium; 3 Laboratory of Morphology and Molecular Pathology, UZ Sint-Raphaël, Leuven, Belgium; University of Nebraska – Lincoln, United States of America

## Abstract

Camelpox virus (CMLV) is the closest known orthopoxvirus genetically related to variola virus. So far, CMLV was restricted to camelids but, recently, three human cases of camelpox have been described in India, highlighting the need to pursue research on its pathogenesis, which has been hampered by the lack of small animal models. Here, we confirm that NMRI immunocompetent mice are resistant to intranasal (i.n.) CMLV infection. However, we demonstrate that CMLV induced a severe disease following i.n. challenge of athymic nude mice, which was accompanied with a failure in gaining weight, leading to euthanasia of the animals. On the other hand, intracutaneous (i.c.) infection resulted in disease development without impacting the body weight evolution. CMLV replication in tissues and body fluids was confirmed in the two models. We further analyzed innate immune and B cell responses induced in the spleen and draining lymph nodes after exposure to CMLV. In both models, strong increases in CD11b^+^F4/80^+^ macrophages were seen in the spleen, while neutrophils, NK and B cell responses varied between the routes of infection. In the lymph nodes, the magnitude of CD11c^+^CD8α^+^ lymphoid and CD11c^+^CD11b^+^ myeloid dendritic cell responses increased in i.n. challenged animals. Analysis of cytokine profiles revealed significant increases of interleukin (IL)-6 and IL-18 in the sera of infected animals, while those of other cytokines were similar to uninfected controls. The efficacy of two antivirals (cidofovir or HPMPC, and its 2, 6-diaminopurine analog) was evaluated in both models. HPMPC was the most effective molecule affording 100% protection from morbidity. It appeared that both treatments did not affect immune cell responses or cytokine expression. In conclusion, we demonstrated that immunodeficient mice are permissive for CMLV propagation. These results provide a basis for studying the pathogenesis of CMLV, as well as for evaluating potential antiviral therapies in an immunodeficiency context.

## Introduction

Camelpox virus (CMLV) is a member of the genus Orthopoxvirus (OPV) of the family *Poxviridae* [1], [2]. In contrast to other OPV members, such as vaccinia virus (VACV), cowpox virus (CPXV) or monkeypox virus, CMLV remains poorly studied, although it is, genetically, the closest virus related to variola virus (VARV) [2]. While other OPVs can infect various hosts, including rodents, zoo animals, monkeys and humans, VARV and CMLV are restricted to a single host, humans for VARV and camels for CMLV, in which they induce a severe disease. Old World (dromedary and Bactrian) camelids have been recognized as the reservoir hosts of CMLV, although New World camelids, such as guanacos, may be experimentally infected [3]. The disease camelpox is endemic in almost every country in which camel husbandry is practiced, and outbreaks have been reported in the Middle East, in Asia, in Africa and in southern parts of Russia and India [4]. The transmission of camelpox is by direct contact or *via* contaminated environment. Of note, arthropod vectors could also be involved in the transmission of the disease [5].

Human cases of camelpox have been described as rare or inexistent [6]–[8]. Indeed, few articles reported individuals with lesions on the arms, or ulcers on the lips and in the mouth (from drinking milk of infected animals), but they all remained unconfirmed [6], [8]. However, recently, camelpox has been described as a possible zoonosis with three human cases identified and laboratory confirmed in India [9]. These camel handlers, in direct contact with camelpox-infected animals, developed skin lesions localized on the fingers and the hands. Identification of CMLV as the causative agent was made (i) based on the detection of camelpox neutralizing antibodies in serum samples of the three suspected cases, (ii) by amplification of a CMLV specific gene (*C18L*), and (iii) by further amplification and sequencing of other genes whose sequences were confirmed to match those of CMLV. These findings should be taken into consideration in the actual context of increasing number of OPV infections in humans and animals. Indeed, the last years, outbreaks of CPXV infections have been identified in European countries and involved humans, pets (rats and dogs) and exotic zoo animals (elephants, mongooses, jaguarundis) [10]–[14]. Also, zoonotic infections of buffalopox have been reported in India [15], and VACV infections are seen in humans, cattle and monkeys in Brazil [16], [17]. It is hypothesized that cessation of smallpox vaccination 30 years ago may have rendered the populations more susceptible to OPV infections, a concept that is supported by the recent finding of a 20-fold increase in human monkeypox incidence in Democratic Republic of Congo [18]. However, camelpox appears largely restricted to camels, and seldom produces clinical disease in humans. Reports of human camelpox cases, either confirmed or not by virological tests, have suggested a mild course of disease even though CMLV is genetically more closely related to VARV than to other OPVs.

In camels, camelpox is a contagious proliferative skin disease with localized or generalized vesicles on the skin and mucous membranes of the lips and the nose [5], [19], [20]. The clinical presentations observed are from mild to severe infections, which may reflect the existence of strains of CMLV of variable pathogenicity [8]. In agreement with previous reports, a recent study showed that young calves and pregnant females were the most susceptible to camelpox [4]. In the same study, the morbidity, mortality and abortion rates were of 30–90%, 1–15% and 80–90%, respectively [4]. Camelpox outbreaks cause not only severe economic losses in camel breeding areas, as camels are used for milk, meat, wool production, transport or racing [21], but also require logistical measures of containment for avoiding the spread in neighboring countries [22].

In this context, laboratories have conducted researches to develop live attenuated (Dubai camelpox vaccine or Ducapox) and inactivated camelpox vaccines [3], [23], [24]. For instance, a single dose of Ducapox, given 6 years prior lethal challenge with CMLV, could protect camels from camelpox disease and related death. However, 6 to 9 month-old camels should receive a booster injection for a full protection [3]. In addition to these prophylactic options, post-exposure therapies could be of interest, particularly in young camels, but they have not yet been investigated *in vivo* due to the lack of small animal models. *In vitro*, ST-246 [Tecovirimat®, 4-trifluoromethyl-N-(3,3a,4,4a,5,5a,6,6a-octahydro-1,3-dioxo-4,6-ethenocycloprop[f]isoindol-2(1H)-yl)-benzamide] potently inhibited CMLV replication in several cell culture models by preventing the production of enveloped virus particles [25]–[27]. Also, several inhibitors of viral DNA polymerase, such as the acyclic nucleoside phosphonate HPMPC [cidofovir or Vistide®, (S)-1-(3-hydroxy-2-phosphoylmethoxypropyl)cytosine] and the 2,6-diaminopurine analog HPMPDAP, have shown potent antiviral activities against CMLV replication *in vitro* [28]. Also, ST-246, HPMPC and CMX-001 (hexadecyloxypropyl-HPMPC) are currently recognized as potent inhibitors of OPVs *in vivo*, as demonstrated in various animal models [29], [30]. ST-246 and CMX-001 are orally available and HPMPC requires intravenous administration. These compounds have been successfully used as emergency investigational new drugs in humans for the treatment of life-threatening VACV infections [31]–[37]. Based on these data, ST-246 and acyclic nucleoside phosphonates could be considered as therapeutic options against natural CMLV infections. However, their further evaluation requires the availability of animal models.

For the past forty years, several studies have supported the narrow host range of CMLV. Indeed, experimental inoculations of CMLV into cattle, sheep, goats, rabbits, guinea pigs, rats, hamsters and adult mice have been unsuccessful [22], [38]–[41]. In one study, monkeys challenged with CMLV strain Etha 78 developed typical pox lesions passing all stages from day 8 to day 18 post-inoculation [39], whereas in another report, monkeys inoculated with CMLV strain CM-G2 did not develop a rash [38]. Suckling and two week-old mice have been shown to be highly susceptible to certain strains of CMLV given intraperitoneally or intracerebrally, with 50% lethal doses varying with the strain used [21], [38], [42]. Five day-old or adult immunocompetent mice have been described resistant to CMLV when inoculated intradermally, albeit such strains caused considerable morbidity in camel herds or generalized pox lesions and death of 10 month-old camels [22], [40], [43]. Nevertheless, the availability of small animal models of CMLV infection would greatly enhance the understanding of CMLV pathogenesis, including its immune evasion mechanisms, and would allow the evaluation of (i) antivirals efficacy and (ii) the virulence of different CMLV strains. Moreover, there are evidences that camelpox might become a zoonosis, and severe forms of the disease should be considered in immunocompromised individuals.

Thus, considering that common adult immunocompetent laboratory mice seemed being resistant to CMLV, we decided to focus on immunodeficient mice. A similar approach has been successfully reported with monkeypox virus [44], [45]. Indeed, most of the laboratory-derived immunocompetent mice appeared resistant to monkeypox virus, while wild-derived inbred immunocompetent strains (CAST/EiJ) and immunodeficient mice, such as SCID BALB/c, C57BL/6 *Stat1^-/-^* and 129 *Stat1^-/-^*, were highly susceptible [44]–[46].

The aim of our work was to investigate whether four week-old athymic nude mice might be permissive for CMLV replication and propagation following intranasal (i.n.) or intracutaneous (i.c.) challenge. We further evaluated if the delivery routes could exert different pathogenic effects, as already reported for other OPVs [Schriewer, J. *et al.*, XVIII International Poxvirus, Asfivirus, and Iridovirus Symposium, Sedona, Arizona, USA, June 5–10, 2010, P8.12], [43], in terms of disease progression and outcome, immune cell recruitment and cytokine production. Our findings demonstrate that the survival and pathogenesis of *nu/nu* mice depends on the route of infection which drives different patterns of immune cell recruitment in the spleen and lymph nodes of infected animals. In addition, the benefits of HPMPC and HPMPDAP treatments given topically or systemically were assessed and it was found that both treatments had an effect on CMLV-induced disease with HPMPC offering 100% protection from morbidity.

## Results

### *nu/nu* mice are susceptible to i.n. and i.c. CML1 infection

In pilot studies, the pathogenicity of CMLV strain Iran (CML1) was first evaluated in 4 to 5 week-old NMRI immunocompetent mice, challenged via the i.n. route with 2.0×10^6^ PFU/mouse. These mice were followed for 70 days after infection and did not show any symptoms or loss of body weight (data not shown). This is in line with published studies describing the lack of virulence of CMLV in immunocompetent mice following intracranial or intradermal inoculation [22], [38]. Also, all of the animals challenged with CMLV seroconverted with a 50% neutralizing antibody titer mean of 1.54±0.23 log_10_ at day 70 post-infection. We then hypothesized that depletion of T cell-mediated responses could facilitate camelpox disease. Indeed, it has been shown that an effective cytotoxic T-lymphocyte response was important for successful OPV clearance, for instance, by inducing interferon-γ (IFN-γ) by T_H_1 cytokines [47]. Athymic nude mice were chosen as they lack thymic T cells, and animals at a young age (4 to 5 week-old, corresponding to an average body weight of 13 to 15 g) were used, since it was shown that susceptibility of mice to CMLV could be age dependent [21]. Based on other animal models of OPV infection, two routes of inoculation were investigated: i.n. and i.c. by light scarification at the lumbosacral region [48]. Various inocula were evaluated in both models to determine the conditions required to reach 100% morbidity or mortality, and these results are presented here. Of note, all animal's deaths recorded in the following experiments were induced by euthanasia based on humane end-points in order to minimize pain and distress, as defined in the materials and methods section.

**Figure 1:**
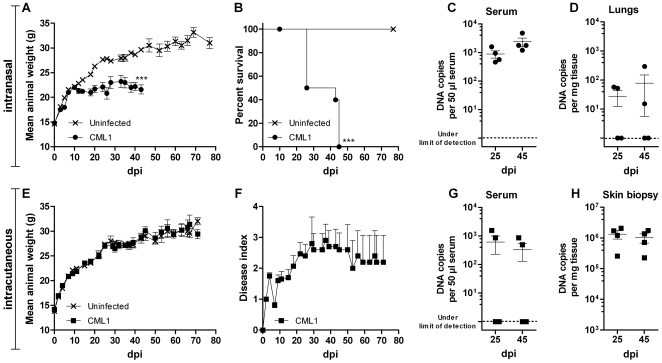
CML1 is pathogenic for athymic nude mice. Body weight evolution (**A, E**), survival curve (**B**), viral titers (**C, D, G, H**) and disease indices (**F**) recorded in *nu/nu* mice infected i.n. (**A–D**) or i.c. by light scarification on the back (**E–H**). (**A–B, E–F**) Ten mice per group (uninfected group and CML1-infected group) were followed for 80 days for their body weight and disease progression. Survival curves (**B**) were built based on i.n. infected animals that were euthanized due to (i) failure in gaining weight and (ii) severe disease when compared to uninfected controls (see materials and methods section). Disease indices (**F**) were attributed as stated in materials and methods. (**C–D, G–H**) Viral loads (*n* = 4 mice per time point) of serum, liver or back skin samples of CML1-infected mice were determined by qPCR (see materials and methods) and plotted on a logarithmic scale as viral DNA copies per 50 µl serum or per mg tissue. Results are presented as means±standard error of the mean (SEM). ***p<0.001: statistic differences in body weight evolution (unpaired *t* test) and in survival (Log-rank test). Results are representative of three independent experiments.

**Figure 2:**
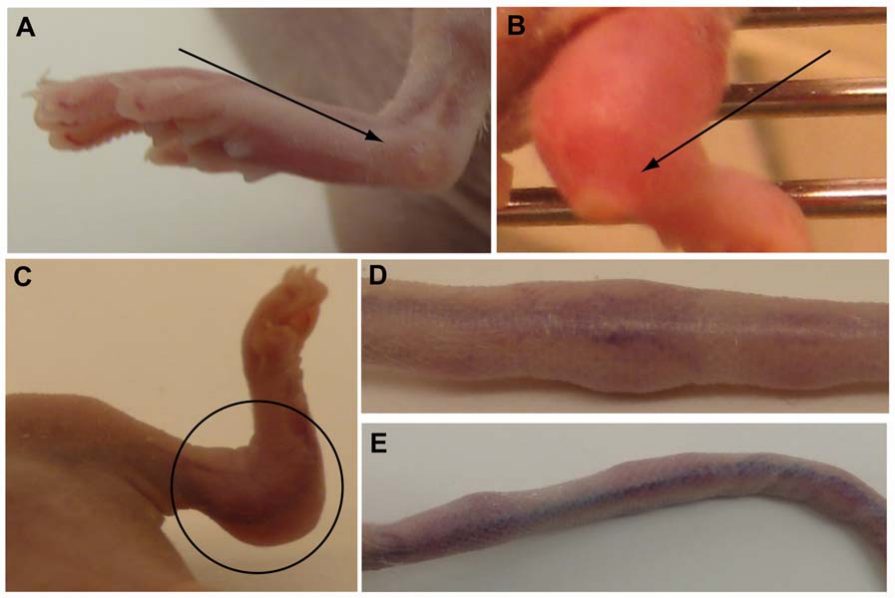
Symptoms observed at day 30 post-infection in *nu/nu* mice infected i.n. with CML1. Note the vesicles localized on the leg (**A and B**), the swelling of the joint of the leg (**B and C**), and the presence of lesions along the tail (**D and E**). Similar symptoms are seen with i.c. infection.

From these experiments, we demonstrated that mice infected i.n. with CML1 at an inoculum of 2.0×10^6^ PFU/mouse failed to gain weight at the same rate as uninfected mice, starting from day 10 post-infection (**Figure 1A** **)**. This was accompanied with severe disease progression, and clinical manifestations of camelpox were seen and consisted of pox-like lesions along the tail and on the legs (starting on day 25 post-infection) while the rest of the body remained lesion-free. Edemas of the joints and of the tail could be observed in some of the animals (**Figure 2**). For these reasons, animals presenting both parameters, i.e. absence of body weight take and severe disease, were euthanized and survival curves could be established. As shown in **Figure 1B**, 100% of the i.n. infected cohort was euthanized by day 45 post-infection. Virus replication could be detected at days 25 and 45 post-infection in the sera and the lungs of, respectively, 100% and 50% of the infected animals by quantitative real-time PCR (qPCR, **Figure 1C and D**). CML1 DNA copies were also detected in liver and spleen at the same time points (data not shown). Importantly, at earlier times, CMLV DNA copies could not be detected in the sera and in various tissues suggesting the spreading of the virus from the initial site of infection (data not shown). The presence of CML1 in skin lesions was confirmed by qPCR analyses of skin lesions (data not shown).

Interestingly, *nu/nu* mice infected with CML1 by scarification (2.0×10^6^ PFU/mouse) exhibited a body weight evolution similar to that of uninfected controls along the 75 days of the experiment (**Figure 1E**). However, i.c. infected mice did develop “pock-like” primary lesions at the site of scarification, which further spread towards the extremities. As shown in **Figure 1F**, animals were scored for severity of lesions that appeared as soon as day 5 post-infection and then extended to the tail and/or legs by day 30 post-infection. In this model, the presence of CML1 was detected in the serum of 50% of the mice, while all skin biopsies exhibited high CML1 DNA copy numbers at days 30 and 45 post-infection (**Figure 1G and H**). At similar time points, animals with CML1-positive serum also demonstrated virus in the spleen and the tail, indicating the systemic spread of the virus (data not shown). This is further supported by the fact that, at earlier times, CMLV DNA copies were only detected in skin biopsies and swabs, and not in other tissues (data not shown).

These results demonstrated that i.n. infection with CML1 resulted in drastic failure in weight gain, which, associated with a severe illness, led to death by euthanasia of the entire cohort. In contrast, CML1 administrated by the i.c. route provoked primary localized lesions, which then disseminated to the tail and/or legs of the mice, but did not affect body weight as compared with uninfected controls. We therefore wondered whether the differences in disease progression seen between the two models of infection could be explained by the circulation of neutralizing antibodies. Indeed, athymic nude mice can mount B cell responses. Seroneutralization experiments were performed with sera (*n* = 4 mice per group) collected at days 25 and 45 post-infection, and at days 30 and 75 post-infection for the i.n. and i.c. infection, respectively. Animals challenged with CML1 via i.n. or i.c. route did not produce neutralizing antibodies. Therefore, CML1 delivery route is of importance in *nu/nu* mice to determine the outcome of the disease.

### Histopathology of *nu/nu* mice infected with CML1

**Figure 3:**
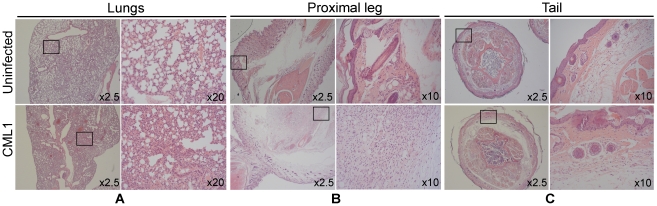
Histological examinations of lungs (A), proximal leg (B) and tail (C) of *nu/nu* mice infected i.n. with CML1. Hematoxylin and eosin-stained tissues are shown at day 45 post-infection for uninfected *nu/nu* mice (upper panels) and animals infected with CML1 by i.n. instillation (lower panels). (**A**) In CML1 infected animals, in comparison with uninfected animals, the lung tissue is characterized by open alveolar spaces, bronchioli without changes, and some dilated vessels in the interalveolar septa. (**B**) The disease induced in CML1 infected animals is also characterized by a massive edema of the muscular compartment of the proximal leg. (**C**) Histopathological changes in the tail upon infection are indentified by a diffuse infiltrate of neutrophils that extends from the deep dermis up to the underlying bone. Magnifications are indicated on each panel, and the 10× or 20× images represent the boxed region of the corresponding 2.5× image.

To further study the two models, histopathology of various tissue biopsies was undertaken. As depicted in **Figure 3A** **, lungs**, at 45 days after i.n. instillation, lung tissue showed the presence of dilated vessels in the interalveolar septa, but no inflammatory cells, and alveolar macrophages were sparse. Also, the epithelium lining the bronchioles was found normal, in comparison with lung tissue of uninfected animals. In addition, the leg was swollen due to massive edema of the muscular compartment; blood vessels showed vasodilatation and the muscle fibers were dissected by a mixed inflammatory infiltrate containing many macrophages; the bone marrow was hypercellular due to hyperplasia of the white cell series (**Figure 3B** **, proximal leg**). Histopathogical examination of the tail showed a swelling due to a diffuse infiltrate of neutrophils that extended from the deep dermis up to the underlying bone; the inflammatory cells infiltrated in and between the muscle fibers, and produced an irregular outline of the cortical bone; increased numbers of osteoblasts and osteoclasts were seen (**Figure 3C** **, tail**).

**Figure 4:**
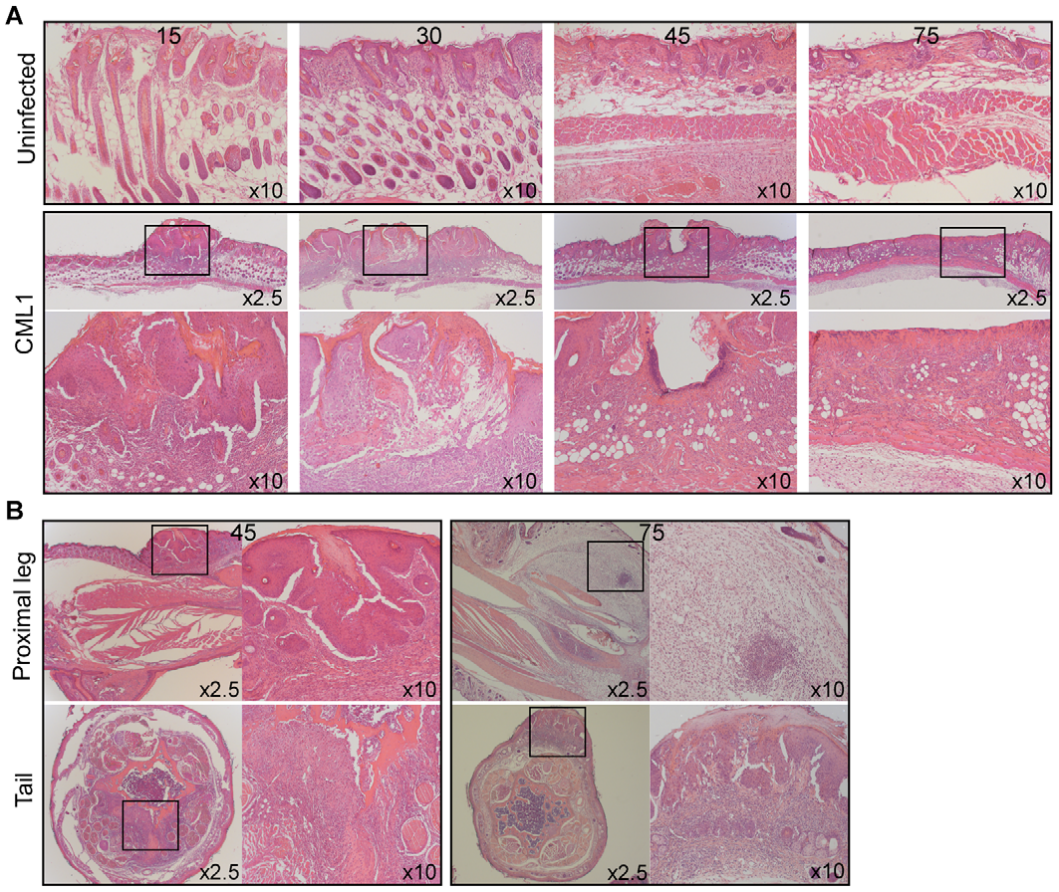
Histology of back skin (scarified-zone), proximal leg and tail in *nu/nu* infected by scarification with CML1. (**A**) Hematoxylin and eosin-stained skin tissues are shown for uninfected and CML1-scarified *nu/nu* mice at 15, 30, 45 and 75 dpi. Inflammation is centered around hair follicles, as well as in the deep muscle, and the hyperplastic epithelium invaginates to form a crater. (**B**) Histological examinations of proximal leg and tail of CML1-scarified *nu/nu* mice are shown at 45 and 75 days after virus infection. Note the epithelial hyperplasia and ulceration in the skin of the leg and of the tail, and the massive edema that extends between individual muscle fibers of the leg at 75 dpi. Magnifications are indicated on each panel, and the 10× images represent the boxed region of the corresponding 2.5× image.

**Figure 4A** shows the back skin from mice infected by scarification. At day 15 post-infection, foci of inflammation around the deep part of follicles, comprised of eosinophils and mononuclear cells, were seen. Fifteen days later, there were several exophytic lesions, covered by hyperplastic squamous epithelium that centrally invaginated to form a crater, filled with parakeratin and inflammatory cells, as well as some exfoliated epithelial cells. The hyperplastic epithelium contained few, scattered dyskeratotic cells. The epithelium was focally eroded and replaced by a crusta. At its base, the lesion was surrounded by a mixed inflammatory infiltrate that extended into the underlying muscular layer. At day 45 post-infection, a partly exophytic, partly endophytic lesion, comprising hyperplastic squamous epithelium that centrally invaginated, was observed. In the stroma around this lesion, a mononuclear inflammatory infiltrate was seen. Finally, at 72 days post-infection (dpi), the skin presented a large erosion in which the squamous epithelium was lost, and replaced by a crusta. In addition, no hair follicles were left in the underlying tissue, and the fatty tissue was broken up by thin collagenous strands containing some mononuclear inflammatory cells.

As shown in **Figure 4B**, similar findings were observed in the skin of the leg and of the tail at 45 dpi. The observations of the leg at day 75 post-infection were comparable to those reported above for the i.n. model. However, in some areas, neutrophils predominated and formed small abcess-like accumulations in which nuclear dust was found. The inflammatory infiltrate encroached focally upon the cortical bone resulting in piecemeal break-down and irregular outline. The tail showed focal epithelial hyperplasia and ulceration, covered by a crusta containing parakeratin; in the epithelium, scattered dyskeratotic cells were seen. At the base of this lesion, a band-like mononuclear inflammatory infiltrate was observed (**Figure 4B**).

### CML1 infection affects innate and B cell responses

The route of CML1 administration appeared to drive the disease outcome. In this context, we speculated that immune responses induced in each model could help understanding CML1 pathogenesis. Athymic nude mice lack thymic T cells, but have potent innate immune, as well as B cell, responses. We determined the changes in the number of macrophages, neutrophils, NK cells and B cells in the spleen and in a pool of draining lymph nodes (DLNs; axillary, inguinal, mesenteric and lumbar) after exposure to CML1, in comparison with non infected animals, by flow cytometric analysis at days 25 and 45 post-infection for the i.n. model, and at days 30 and 75 post-infection for the i.c. model. Of note, we observed a severe enlargement of the lumbar lymph nodes in both models, and scarified-animals also exhibited moderate enlargement of inguinal and axillary lymph nodes, as seen at days 30 and 75 post-infection.

**Figure 5:**
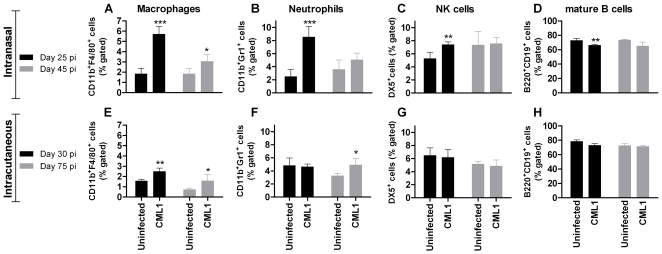
Identification of changes in immune cell populations from spleen of *nu/nu* mice exposed to CML1. Mice (*n* = 4 per group) were challenged i.n. (**A–D**) or i.c. (**E–H**) with PBS (uninfected) or with CML1 at a dose of 2.0×10^6^ PFU. At 25 and 45 dpi for the i.n. model or at 30 and 75 dpi for the i.c. model, spleen were harvested and CD11b^+^F4/80^+^ macrophages, CD11b^+^Gr1^+^ neutrophils, DX5^+^ NK cells and B220^+^CD19^+^ mature B cells were identified by FACS. Results are presented as percent gated cells. Bars represent mean±SD, standard deviation (*n* = 4). ***p<0.001; **p<0.01 and *p<0.05: CML1 differs significantly from uninfected group as determined by unpaired *t* test; representative of two independent experiments.

Analysis of splenocytes revealed a significant and sustained recruitment of CD11b^+^F4/80^+^ macrophages and a strong, but transient, increase of CD11b^+^Gr1^+^ neutrophils in the spleen after i.n. CML1 inoculation (**Figure 5A and B**). A comparable pattern of macrophage increase was seen after i.c. infection, whereas the neutrophil population only rose at 75 dpi (**Figure 5E and F**). In terms of NK cell response, a significant and temporary increase in DX5^+^ subset was observed at 25 dpi following i.n. challenge. In contrast, no significant changes in NK cell populations were noticed after i.c. inoculation (**Figure 5C and G**). After i.n. infection, B220^+^CD19^+^ B cell subset was slightly decreased at days 25 and 45 post-infection, and this trend was also viewed in the i.c. model (**Figure 5D and H**). It has to be mentioned that the decreased trend of the B220^+^CD19^+^ population was not statistically significant in other independent experiments. Our results suggest that the recruitment of neutrophils, DX5^+^ NK cells and B220^+^CD19^+^ cell population differ to some extent between the two models, while macrophages are similarly recruited.

**Figure 6:**
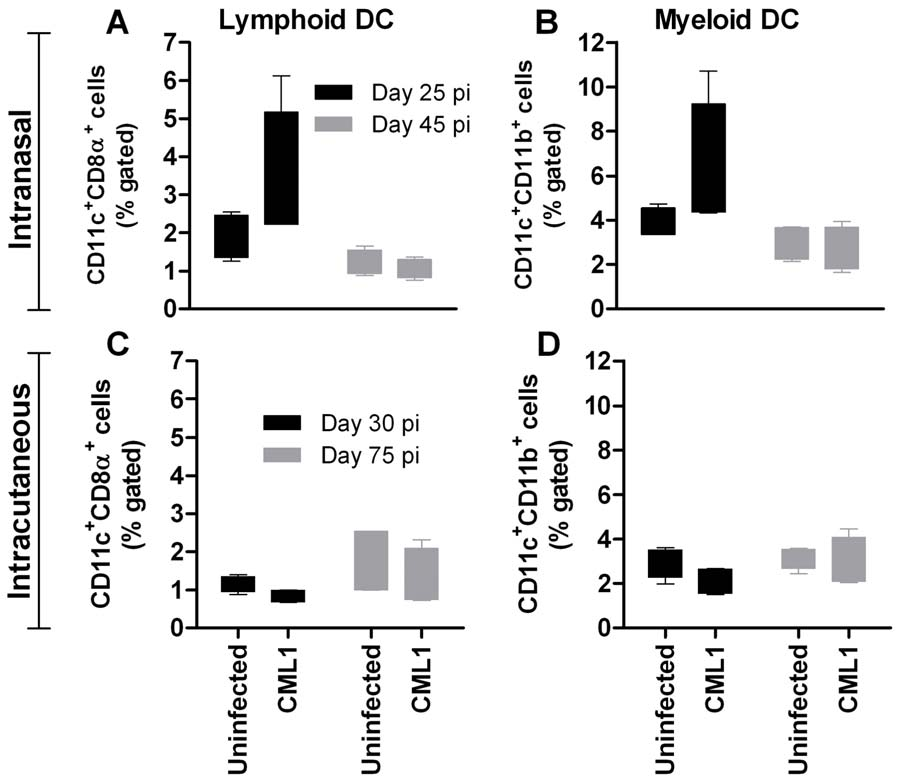
DLN responses to CML1 in *nu/nu* mice following i.n. or i.c. infection. Mice received PBS (uninfected) or 2.0×10^6^ PFU of CML1. At 25 and 45 dpi for the i.n. model (**A–B**) or at 30 and 75 dpi for the i.c. model (**C–D**), DLNs (pool of inguinal, axillary, mesenteric and lumbar lymph nodes) were collected and the CD11c^+^CD8α^+^ lymphoid and CD11c^+^CD11b^+^ myeloid DCs were identified. The magnitude of DC responses is depicted as percent gated cells plotted on box- and whiskers-graphs. Bars represent Min to Max whiskers (*n* = 4 individual mice for each group). Results were submitted to unpaired *t* test but the differences between the groups were not significant.

Analysis of the DLNs i.n. and i.c. inoculations revealed no changes in the levels of DX5^+^CD3^-^ NK cells and of B220^+^CD19^+^ mature B cells in both models, compared with the uninfected controls (data not shown). In contrast, after i.n. challenge, the amounts of CD11c^+^CD8α^+^ lymphoid and CD11c^+^CD11b^+^ myeloid dendritic cells (DCs) appeared increased at 25 dpi, in comparison with uninfected *nu/nu* mice (**Figure 6A and B**). However, these differences were not statistically significant but were seen in two independent experiments. At 45 dpi, the levels of the two DC subsets were comparable to those of the controls. In contrast, at 30 dpi, there was a little trend of decreased amounts of lymphoid and myeloid DCs in DLNs of mice inoculated by the i.c. route compared with the controls, while at 75 dpi, both DC populations remained constant (**Figure 6C and D**). Therefore, both routes of inoculation only induced small changes in the distribution of the various cell populations (i.e., DCs, NK and B cells) found in the DLNs.

### CML1 infection alters cytokine profiles in the sera

**Figure 7:**
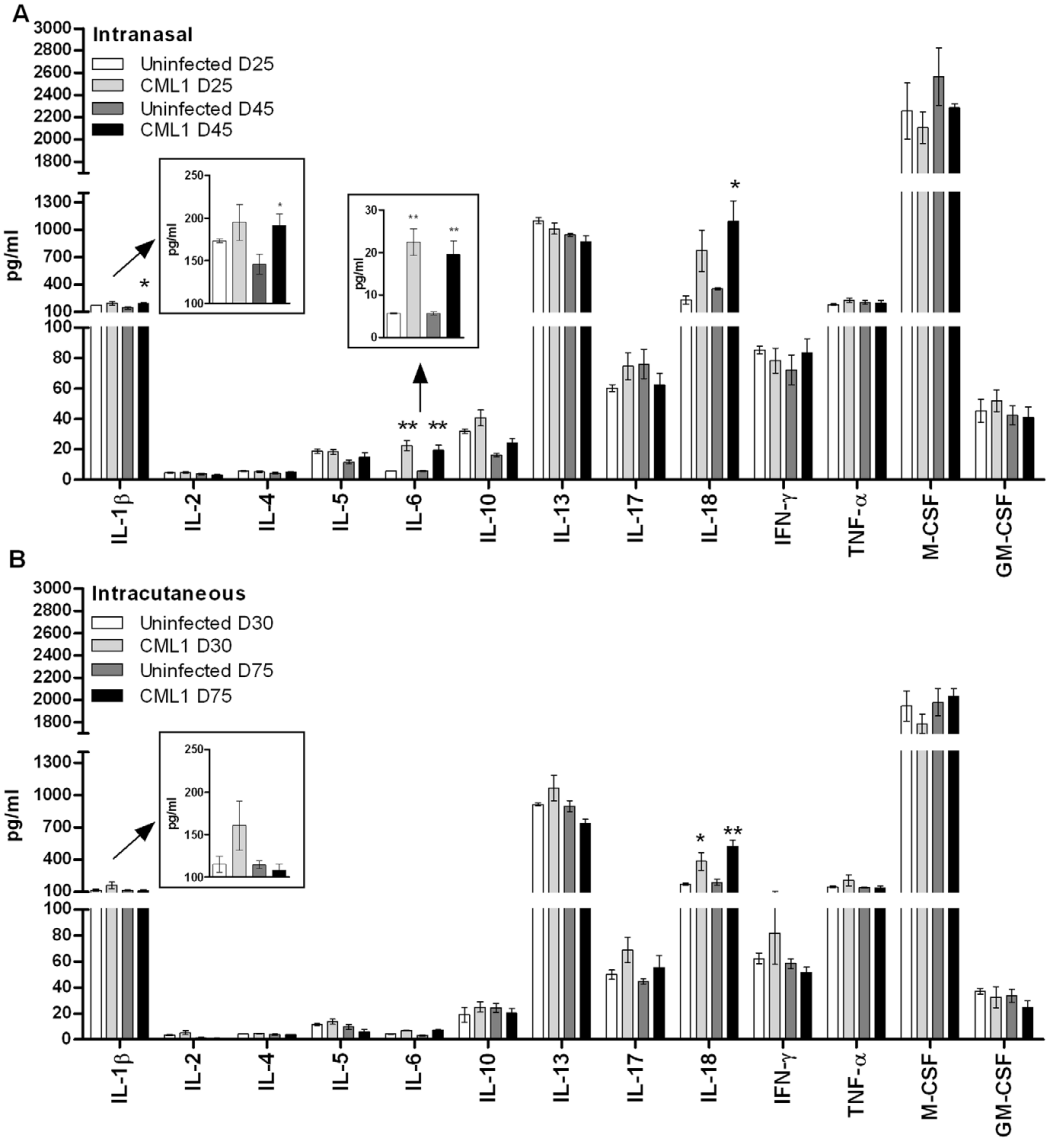
Cytokine production in the sera of *nu/nu* after exposure to CML1. Sera from mice inoculated i.n. (**A**) or i.c. (**B**), isolated at two time points after exposure to PBS or to CML1, were used to measure cytokine levels by ELISA. Data are the average±SEM (*n* = 4 mice for each group). ***p<0.001; **p<0.01 and *p<0.05, CML1 versus the corresponding values of the uninfected group by unpaired *t* test. Boxed regions represent a zoom-in of the corresponding field, i.e., IL-1β and IL-6.

A number of cytokines have been shown to play important roles in the virulence or in the resolution of acute infection of OPVs [49]–[51]. Thus, to better understand the contrast seen between the i.n. and i.c. route of infection, we aimed to examine cytokine responses in the serum of animals exposed to CML1. For that purpose, sera were collected on days 25 and 45, and on days 30 and 75 after virus exposure for the i.n. and i.c. model, respectively. Four animals per group and per time point were used. As shown in **Figure 7A**, i.n. CML1 challenge resulted in significant increase of interleukin (IL)-18 at day 45 post-infection as compared with uninfected controls; though this trend was already seen at 25 dpi. A 4-fold increase in the levels of IL-6 was recorded upon i.n. infection, albeit only low levels of IL-6 were detected (mean levels of 20 pg/ml), compared with uninfected controls. This increase has been further confirmed in an additional ELISA assay (see materials and methods) which showed mean IL-6 levels of 71±34 pg/ml and of 65±23 pg/ml in the sera of i.n. infected mice at, respectively, 25 and 45 dpi, while the uninfected animals had IL-6 levels under the limit of detection. IL-1β appeared very slightly up-regulated after CML1 exposure, but precaution should be taken to interpret this result as the significance is based on a *p* value of 0.0456. All the other cytokines evaluated in the i.n. model had levels similar to those of the uninfected animals.

Interestingly, a similar cytokine profile was seen in the i.c. model with IL-18 levels being boosted in response to CML1 scarification, while the levels of the other cytokines were in the range of the controls, including IL-6 (**Figure 7B**). This result, further confirmed with an ELISA assay, highlighted the discrepancies in IL-6 levels seen between the two models, although precaution should be taken as the IL-6 levels observed in the i.n. model were low.

### Systemic HPMPC, and to a lesser extent HPMPDAP, protect *nu/nu* mice against severe disease development after i.n. challenge with CML1

**Figure 8:**
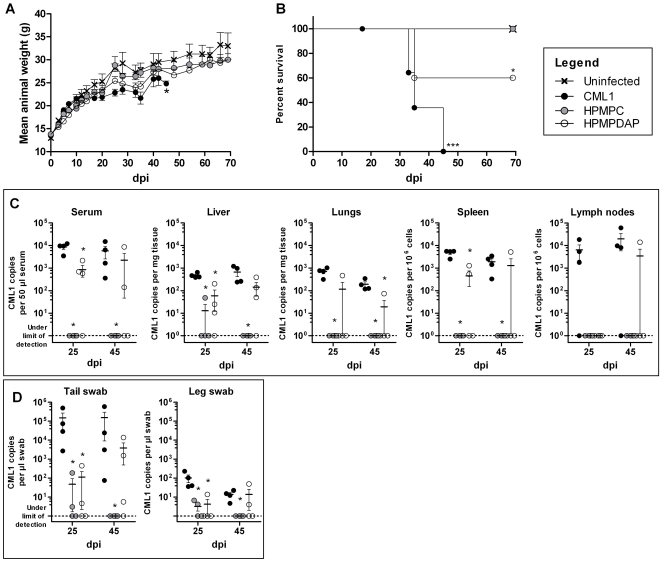
HPMPC protects against lethal CML1 i.n. challenge, whereas HPMPDAP showed an intermediate protection. *Nu/nu* mice were inoculated i.n. with PBS (uninfected group) or with CML1 at a dose of 2.0×10^6^ PFU (CML1, HPMPC and HPMPDAP groups). HPMPC and HPMPDAP groups were treated intraperitoneally for 3 days, starting the day of infection, with 50 mg/kg of HPMPC or HPMPDAP, respectively; uninfected and CML1 groups received a similar treatment regimen but with PBS. Animals were monitored for 70 days for body weight (**A**). Survival curves (**B**) were built based on i.n. infected animals treated or not that were euthanized due to (i) failure in gaining weight and (ii) severe illness in comparison with uninfected controls (see materials and methods). Viral loads in the serum and various organs (**C**) and in skin swabs of tail and leg (**D**) were determined by qPCR at days 25 and 45 post-infection. Data are mean±SEM. Statistical analyses were performed as described in materials and methods. Results are representative of two independent experiments.

To investigate the feasibility of using the i.n. model for the evaluation of antivirals, we assessed the efficacy of HPMPC and of HPMPDAP. The choice of HPMPDAP was based on its 10-fold higher antiviral activity against CML1 in cell culture, compared to HPMPC [28]. Four-week old mice were infected i.n. with 2.0×10^6^ PFU of CML1, and treated intraperitoneally for 3 days, once per day, with 50 mg/kg of HPMPC and HPMPDAP or with PBS (placebo). As shown in **Figures 8A and 8B**, body weight curve of animals infected with CML1 and non-treated significantly diverged from that of the uninfected group from day 17 post-infection, and all animals had to be euthanized between days 32 and 45 post-infection based on our defined end-points (see materials and methods). In contrast, HPMPC treatment protected mice from changes in body weight and severe illness (**Figures 8A and 8B**). Animals infected and treated with HPMPDAP showed significant failure in weight gain, compared to uninfected group, which started at day 5 post-infection, and 40% of these mice did develop severe disease requiring euthanasia (**Figure 8A**). From the entire HPMPDAP cohort, 60% of the animals survived CML1 challenge, as seen at day 70 post-infection (**Figure 8B**).

As depicted in **Figure 8C and D**, virus dissemination was confirmed in the serum, liver, lungs, spleen and lymph nodes of all animals of the CML1 group, and the viral DNA loads showed that animals were similarly infected. The presence of viral genome in skin lesions of the tail or leg was also validated. Under HPMPC treatment, CML1 viral genome remained undetected in the serum, lungs, spleen and lymph nodes at days 25 and 45 post-infection. However, at 25 dpi, one mouse did show virus DNA copies in the liver, and two mice had positive swabs of the tail and of the leg, but these viral DNA loads were still significantly lower than those seen in the CML1 group. At day 25 post-infection, HPMPDAP treatment significantly reduced viral DNA loads in the serum, liver, spleen and swabs compared to those of CML1-group. Also, at that day, the only organs that remained free of CML1 under HPMPDAP therapy were the lymph nodes. At 45 dpi, depending on the organ analyzed, one to three out of four of the euthanized mice exhibited CML1 DNA copies. This suggests that systemic administration of HPMPDAP for 3 days was not as effective as HPMPC for preventing CML1 dissemination in *nu/nu* mice.

### Topical HPMPC and HPMPDAP affect CML1 dissemination in *nu/nu* mice following i.c. infection

**Figure 9:**
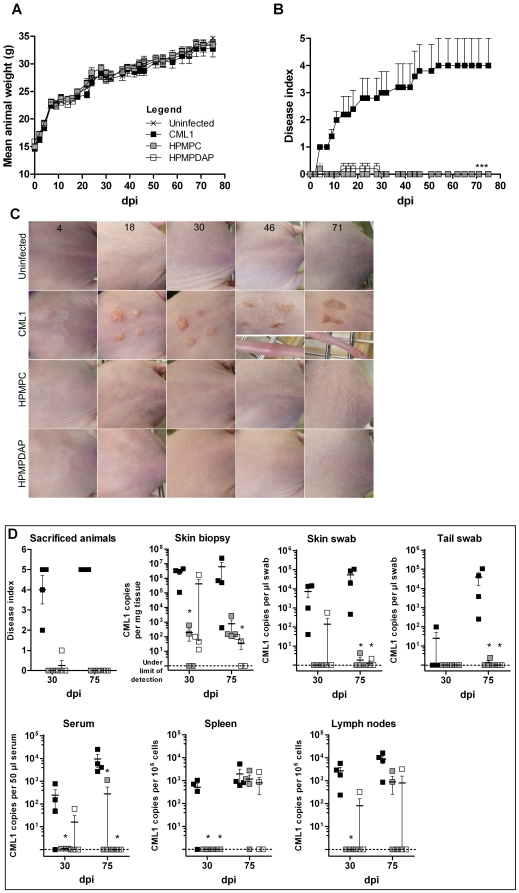
Effects of topical HPMPC and HPMPDAP against CML1 propagation in cutaneously infected *nu/nu* mice. Animals were scarified with PBS (uninfected group) or with CML1 at a dose of 2.0×10^6^ PFU (CML1, HPMPC and HPMPDAP groups). Topical application of 1% HPMPC- and HPMPDAP-cream started the day of infection for 5 days, once per day. Uninfected and CML1 groups were treated similarly with a placebo-cream. Animals were monitored for 75 days for body weight (**A**, *n* = 5), lesions development (**B–C**), and viral loads (**D**). **B**; Disease indices or scores were given as stated in materials and methods. Data are mean±SEM (*n* = 5). **C**; photos were taken at 4, 18, 30, 46 and 75 dpi, and show the evolution of the primary lesions and their spread to the tail. **D**; the disease index of the sacrificed mice (*n* = 4 per group) is shown. Viral loads in various tissues and swabs were determined by qPCR at days 30 and 75 post-infection. Data are mean±SEM. For statistical analyses, see materials and methods. Results are representative of two independent experiments.

We further wanted to determine whether the scarification model could be useful for evaluating the antiviral efficacy of cream-based therapeutics. The efficacy of 1% HPMPC-cream has already been shown against cutaneous VACV or CPXV infections in immunodeficient mice [52], [53]. Here, in addition to an HPMPC-cream, we also evaluated the antiviral activity of an HPMPDAP-cream against CML1-induced disease. Athymic nude mice were inoculated with CML1 by light scarification at the lumbosacral region. Topical treatments of 1% HPMPC- or HPMPDAP-cream were given once daily for 5 days, starting the day of infection, and mice were followed for 75 days after virus contact. As expected, no changes in body weight were seen between the different groups (**Figure 9A**). In addition, HPMPC and HPMPDAP therapies abolished lesion development at the site of scarification and propagation to the tail and/or leg through the 75 days of monitoring, as demonstrated in **Figures 9B and C**. It has to be noticed that the 1%-HPMPC formulation could cause skin irritation, which was not observed in the placebo- or HPMPDAP-group (data not shown).

Viral loads in various samples were then determined at days 30 and 75 post-infection. For that purpose, four mice of each group were chosen at random, sacrificed at both time points and their scores (disease indices) were recorded. As shown in **Figure 9D**, all animals from the CML1-cohort had mean scores of 4 and 5, demonstrating the severity of the lesions. It has to be mentioned that the back lesion of one mouse killed at day 75 post-infection was almost healed but this animal still had severe lesions on the tail and swollen joints. The HPMPC-group had no signs of disease (score of zero). Interestingly, a score of 1 was reported in one animal of the HPMPDAP-group at day 30 post-infection, while the others remained disease free. As shown in **Figure 9D**, animals belonging to the CML1-cohort exhibited intermediate to high viral loads among the various samples. Per time points and per samples, CML1 copy numbers were nicely grouped to each other, with the exception of spleen and serum samples at day 30 post-infection. At 30 dpi, viral loads found in skin biopsies were diminished approximately by 10,000-fold after HPMPC treatment, compared with the CML1-group, which showed an average viral DNA copies of 5×10^6^ (**Figure 9D**). HPMPC treatment resulted in undetectable levels of CML1 at 30 dpi in swabs from skin and tail, and in sera, spleen and lymph nodes, compared to those of the CML1-cohort. At later time point, the antiviral efficacy of HPMPC treatment was still seen with a strong reduction or inhibition of viral loads in the skin biopsies, skin and tail swabs and serum. However, HPMPC only delayed viral dissemination to the spleen and the lymph nodes at day 75 post-infection, since, respectively, 50% and 75% of the animals had viral loads similar to those of the CML1-group.

HPMPDAP topical treatment afforded strong reductions in viral DNA loads in the various samples recovered in 3 out of 4 animals at day 30 post-infection. At this time point, the mouse that showed high viral DNA copies, not only in the skin biopsy, but also in most of the other samples examined (i.e., skin swab, serum and lymph nodes), corresponded to the one reported with a disease index of 1 (**Figure 9D**). At 75 dpi, statistically significant diminutions in viral DNA loads, reaching sometimes undetectable levels, were seen in the skin biopsies, swabs and sera recovered from mice under HPMPAP therapy. Similarly to HPMPC, topical HPMPDAP provided only partial effect in reducing titers in the spleen and lymph nodes. As a general conclusion, both topical treatments inhibited the development of primary cutaneous lesions and of satellite lesions as well, at 30 and 75 dpi, but failed to clear the entire cohorts at later time points from CML1 spread to the spleen and the lymph nodes.

### Immune responses and cytokine production following HPMPC and HPMPDAP treatments are similar to those of the uninfected animals

Finally, in order to explore whether HPMPC or HPMPDAP treatments could have an effect on the immune system to help for viral clearance, we characterized the immune cell populations in spleen and DLNs (by FACS analysis) and compared cytokine induction in sera (by ELISA) among the different cohorts. During the animal experiments in which the antiviral activities of HPMPC and HPMPDAP were assessed, samples were collected and used for these analyses. FACS experiments performed on spleen and DLNs revealed that the levels of immune cells, such as macrophages, neutrophils, NK, B, lymphoid and myeloid DC cells, in the infected-treated cohorts were comparable with those of uninfected animals. Likewise, levels of cytokines were comparable between infected-treated animals and uninfected mice. Thus, at the time-points investigated, the treated animals exhibited immune and cytokine responses that were similar to those of naïve animals.

## Discussion

In this report we described the establishment and the characterization of two immunodeficient mouse models of CMLV infection. The choice of an immunodeficient model was based on the literature [38], [41] and on our data confirming that four to five week-old immunocompetent mice were resistant to i.n. CMLV infection and that the entire cohort had neutralizing antibody titers against CMLV. This avirulent phenotype could be circumvented by using severe combined immunodeficient (SCID) mice, which lack B and T cells (data not shown), and athymic nude mice, which lack a normal thymus and thus functionally mature T cells. We observed that i.n. or intraperitoneal administration of CMLV to SCID mice induced 80% mortality between 20 to 30 dpi at a dose of 3×10^5^ and 4×10^6^ PFU, respectively (data not shown). Further experiments performed in athymic mice showed that CMLV given i.n., i.c. or intraperitoneally rendered animals sick. Disease appearance and CMLV-related death (by euthanasia) in *nu/nu* mice were observed later to what was seen with the SCID mice. Therefore, the involvement of B cell responses might be then hypothesized for slowing down camelpox disease progression. We focused our works on *nu/nu* mice, which are partially immunosuppressed, and decided to investigate the two routes of infection that resemble those occurring in the natural host.

We showed that CMLV given by the i.n. and the i.c. route induced the development of camelpox disease in *nu/nu* mice. In both models, the clinical signs of camelpox disease were noticeable with appearance of lesions along the tail, starting from its base towards the extremity, together with edemas of the tail and/or of the joints of the feet and legs. The outcome was different depending on the model of infection, one altering the body weight evolution and leading to death by euthanasia while the other model not. The progression and severity of camelpox disease was measured (i) in the i.n. model by the absence of body weight gain associated with severe illness which justified the euthanasia of the animals and (ii) in the i.c. model by the development of cutaneous ulcerations. Also, monitoring of the disease was possible by performing qPCR on various tissues, body fluids and swabs. Consequently, the two models offered suitable features for enabling the visual and molecular evaluation of antiviral drug efficacies.

In general, the disease observed upon CMLV infection in *nu/nu* mice was prolonged, in contrast with what is seen with pathogenic OPVs such as VACV strain Western Reserve (VACV-WR). Indeed, at doses of virus ranging from 10^4^ to 10^6^ PFU, i.n. infection with VACV-WR resulted in death of all *nu/nu* mice by day 9 post-infection [54], while mortality by euthanasia was observed between days 32 to 45 post-infection with CMLV (dose of 2×10^6^ PFU). This difference in pathogenicity can also be seen in the i.c. model in which CPXV strain Brighton (CPXV-Br) or VACV-WR inoculated to *nu/nu* mice at a dose of 10^4^ PFU induced death of the entire cohort at day 12 after virus exposure (our unpublished data), while animals scarified with CMLV (dose of 10^4^ PFU) did not exhibit signs of disease. However, increasing the dose of CMLV in the i.c. model caused development of lesions at the site of scarification and their propagation to the tail and/or legs. Therefore, in comparison with other pathogenic OPVs, CMLV exhibits an intermediate phenotype with an attenuated degree of virulence in mice. However, CMLV virulence appears to be similar to that seen with vaccine strains of VACV, such as Lederle and Lister, in immunodeficient mice. Indeed, VACV strain Lederle administrated i.n. to *nu/nu* mice at a dose of 2×10^6^ PFU led to death of the animals between days 23 to 33 post-infection, while a 100-fold lower virus dose induced 25% mortality (our unpublished data). Furthermore, i.c. inoculation of VACV strain Lister (dose of 5×10^5^ PFU) to athymic nude mice resulted in death at day 34±7 post-infection [55].

In terms of histopathology, examination of cutaneous lesions described in the i.c. model revealed epithelial hyperplasia, inflammation centered on the hair follicles that extended into the deep muscle in some cases, and development of tumor-like lesions. These features could resemble the histological descriptions of skin lesions found not only in animals infected i.c. with other OPVs such as ectromelia virus (ECTV) [48], but also in humans with VARV, monkeypox virus and molluscum contagiosum virus [56]–[58]. In the case of the i.n. model, lung histology demonstrated the presence of dilated vessels in the interalveolar septa without signs of inflammation. More pronounced respiratory infections of mice have been described following i.n. route with ECTV, VACV and CPXV which provoked lung inflammation, necrotizing bronchopneumonia and bronchiolitis, respectively [48]. However, our observations are comparable to those showing that lethal i.n. CPXV infection performed with a volume of virus of 5 µl led to absence of histological changes of the lungs, while a volume of 50 µl resulted in pneumonitis [50]. We hypothesized that the 10 µl volume used for CMLV infection might have confined pathological changes to the upper respiratory tract.

From our experiments, it appeared that (i) T cell responses were essential for controlling CMLV infection in immunocompetent animals, which is in agreement with published data on other OPVs [59]–[61], (ii) the site of virus entry might give different immune responses which could be responsible for different disease outcome, and (iii) eventually, the genetic background of CMLV itself may help understanding its attenuated virulence phenotype, such as the absence or presence of specific immunomodulatory genes or of host range genes from CMLV genome. Also, our findings with CMLV might help to understand some of the mechanisms used by VARV to evade the host immune system since both viruses share common biological properties including their closely related genomic sequences, their single host species and their poor pathogenicity in adult immunocompetent mice [38].

As a first step to understand the differences seen in the pathogenicity induced by CMLV when it was given i.n. or i.c., we characterized the immune responses induced by CMLV challenge, and the cytokines produced in sera as well. It has indeed been shown that following i.n. or intradermal VACV-WR infection in BALB/c mice, marked differences were seen in the pathogenesis and in the cellular inflammatory response [62]. O'Gorman and colleagues have recently evidenced that VACV immunization induced a TLR2- and IL-6-dependent STAT3 pathway, while lethal ECTV infection did not activate STAT3 [63]; albeit this was observed using two different viruses. Finally, Abadie *et al.* have nicely shown that modified VACV Ankara (MVA) administrated via the intradermal or intramuscular route could recruit antigen-presenting cells (APCs) in a different manner, priming different T cell responses and thus shaping different quality of cytokine-producing virus-specific T cells [64]. Therefore, the route of OPV infection, including CMLV, can result in different pathogenicity by inducing distinct immune responses [Schriewer, J. *et al.*, XVIII International Poxvirus, Asfivirus, and Iridovirus Symposium, Sedona, Arizona, USA, June 5–10, 2010, P8.12] [43], [65].

Nude mice, even though lacking functional T cells, can mount innate immune and B cell responses. At day 25 post-infection, i.n. infection resulted in substantial flow of macrophages, neutrophils and DX5^+^ NK cells in the spleen, while the pool of B cells decreased. Moreover, at the same time, recruitment of myeloid and lymphoid DCs was noticed in the DLNs. Intracutaneous infection, in contrast, resulted in an influx of macrophages in the spleen at days 30 and 75 post-infection, while slight accumulation of neutrophils was observed only at late time point, and no change, compared to uninfected controls, was found in the DLNs. In conclusion, the two models recruited or diminished different cell population pools in response to CMLV, even though they exhibited comparable massive macrophage invasion. In both models, the presence of neutralizing antibodies was not detected.

We further examined the cytokines released in the sera of *nu/nu* mice after i.n. or i.c. challenge. Poxviruses possess various immune evasion strategies to counteract host cell-mediated immunity. To this end, CMLV expresses for example proteins that bind IFN-γ, IFN-α/β, CC chemokines, tumor necrosis factor and IL-1β [2]. Cytokines such as IL-2, -4, -5, -10, -13, -17 and IFN-γ, tumor necrosis factor (TNF)- α, macrophage and granulocyte-macrophage colony stimulating factor (M-CSF and GM-CSF, respectively) in infected animals were in the same range as in uninfected controls. In our hands, i.n. inoculation provoked a very slight increase in IL-1β level at 45 dpi. However, IL-1β may be inactive since CMLV possesses two ways for inhibiting IL-1β activity. The first way could be the production of a viral soluble receptor for IL-1β, encoded by three CMLV genes *CMLV193*, *194* and *196* which are multiple regions of the VACV B16R IL-1 binding protein; although there are no proofs that these small open reading frames are functional [1]. The second way is the production of a caspase 1 inhibitor, known as SPI-2 (*CMLV191* gene homolog to VACV *B13R* gene [1]), which blocks the cleavage of proIL-1β into IL-1β [1].

Intranasal CMLV inoculation of immunocompromised mice led to significant increases of IL-6 levels by approximately 4-fold. Nevertheless, this increase remains much lower than that observed in immunocompetent mice upon lethal VACV or CPXV infection for which, in comparison with uninfected controls, a 60-fold increase in IL-6 concentrations has been recorded [50], [66]. It is recognized that IL-6 deficient animals are highly susceptible to OPV infection demonstrating its role for surviving an OPV infection [49], [63]. However, as hypothesized by Smee *et al.*, the hyper-induction of IL-6 and its sustained levels might be deleterious for the host, counteracting the beneficial role of timely release cytokines for helping virus clearance [50].

Both routes of CMLV inoculation induced an increase in IL-18 levels. These findings may reflect our FACS data since this pro-inflammatory cytokine can be produced by macrophages. Interestingly, although the IL-18 increase was measured after CMLV contact, IFN-γ levels in CMLV-infected animals were similar to those of uninfected animals. In contrast to VACV, VARV or ECTV, CMLV does not encode for an IL-18 binding protein, although IL-18 is a key pro-inflammatory cytokine, originally identified as an IFN-γ-inducing factor. IL-18 has been shown to induce Th1 responses, NK cell activation and T cell proliferation, and to induce host-derived IFN-γ [67]. OPV IL-18 binding proteins have been shown to promote virulence by decreasing IFN-γ levels, and NK and T cell activities as well [68]. The authors have also described that IL-18 was still produced after *in vivo* VACV infection, demonstrating that IL-18 binding protein did not affect levels of IL-18 in the lungs. According to these findings and our data showing the recruitment of DX5^+^ NK cells in the spleen and increased IL-18 levels, we expected to see elevated levels of IFN-γ in the sera (although we might have failed to detect it because our first time point was 25 dpi). In addition, Alcami *et al.* described that the soluble receptor of IFN-γ produced by CMLV could, *in vitro*, bind and inhibit the biological activity of human, bovine and rat IFN-γ, but not mouse IFN-γ, favoring the hypothesis that IFN-γ could have been induced in response to CMLV [69]. We can also speculate that the absence of IFN-γ production could be explained by the activity of the *CMLV097* gene product. The *CMLV097* gene is homolog of the VACV *H1L* gene that encodes for a dual-specificity protein tyrosine phosphatase, which has been shown to dephosphorylate STAT1 and thus prevent the induction of IFN-γ [70].

From these data, it appears that the differences in outcome upon i.n. and i.c. challenge do not seem being related to difference in cytokine profiles. However, in this study, FACS and cytokine analyses were performed late after virus exposure. In further experiments, earlier, as well as closer time points should be considered in order to evaluate whether striking discrepancies between the two models exist and to investigate whether a transient induction of IFN-γ may occur. Moreover, early immune responses should be studied not only in *nu/nu* mice, but also in CMLV-resistant immunocompetent mice, with the aim to identify the immune events trigger by these two types of infection.

To our knowledge, this is the first time that the benefit of an antiviral therapy has been evaluated *in vivo* against CMLV. Two compounds among the acyclic nucleoside phosphonate family were tested, but HPMPC was used as a proof of concept for validating our *in vivo* models to assess therapeutics. Indeed, HPMPC has already proved its anti-poxvirus activity *in vivo* for several years now and is widely used as reference compound in animal studies involving poxviruses [71]. In our hands, systemic HPMPC (50 mg/kg for 3 days once a day) protected *nu/nu* mice in the i.n. model from camelpox disease and related death by euthanasia. Furthermore, following HPMPC treatment, CMLV was cleared from the serum, liver, spleen, lungs and lymph nodes, and no signs of sickness were seen at the end of the experiment. In contrast, although promising from the *in vitro* studies, HPMPDAP exhibited a lower antiviral efficacy, compared to its parent molecule HPMPC, since HPMPDAP protected 60% of the animals from death by euthanasia, and 25% to 50% from systemic spread of the virus, as shown by viral DNA loads. In addition, signs of toxicity were seen following systemic HPMPDAP treatment in *nu/nu* mice aged of four to five weeks, while this was not reported in adult NMRI mice [72]. Regarding the i.c. model, topical treatment with 1% HPMPC abolished primary cutaneous lesions and inhibited the development of lesions on the tail and/or legs, as well as the edemas of the joints. In agreement with that, viral loads in skin biopsies were reduced by 10,000-fold and circulating CMLV was not detected in the sera, spleens and lymph nodes at day 30 after virus exposure. At the end of the experiment, no recurrent lesions were visualized in the treated group, although a systemic presence of CMLV was found in most of the animals. These results demonstrated that HPMPC-cream slowed camelpox disease progression and was not able to counteract the systemic dissemination of CMLV. Smee and colleagues reported comparable observations with VACV, although their model in cyclophosphamide immunosuppressed mice was lethal [53]. The protection afforded by a 1%-HPMPC-cream against VACV-WR reduced the severity and the spread of the lesions, and delayed death. Additionally, topical administration of 1% HPMPC-DMSO solution in *nu/nu* mice scarified with VACV strain Lister, a vaccinal strain, offered a complete protection from disease development and death, although viral loads in vital organs were not reported [55]. Comparable conclusions could be drawn with the HPMPDAP-cream, 100% protection was given against disease appearance and propagation, while systemic spread of CMLV to vital organs was delayed but not stopped.

In conclusion, we developed murine models of CMLV infection, and defined the immunologic parameters induced by CMLV which represent the basis needed for the further study of CMLV pathogenesis and for antiviral drug testing. Moreover, CMLV strains of different pathogenicity might exist [21] and these models could serve for the evaluation of their virulence. Our findings demonstrate also that these models could serve for a better understanding of the immune evasion mechanisms of CMLV, which were until now hampered by the absence of *in vivo* models. The immune modulation offered by CMLV was often characterized by cloning a CMLV gene of interest into a VACV backbone genome [73], [74]. We provide here novel tools for avoiding genetic manipulations between two OPV species, VACV, which can be pathogenic for humans, and CMLV, which induces a localized to generalized and lethal disease in camels.

## Materials and Methods

### Cells and virus

CMLV strain Iran (CML1, kindly provided by H. Meyer, Bundeswehr Institute of Microbiology, Germany) was used in these studies [40]. Human embryonic lung (HEL) fibroblast cells were grown in Earle's minimum essential medium (MEM Earle's, Invitrogen™, Merelbeke, Belgium) containing 5% fetal calf serum (FCS), 0.2% serum substitute for animal cell culture (Ultroser^®^G, PALL Life Sciences, Cergy-Saint-Christophe, France), 1% HEPES, 1% non-essential amino acids, 1% sodium pyruvate, and 1% penicillin/streptomycin/glutamine (Invitrogen™) at 37°C under a 5% CO_2_ atmosphere. Virus was grown and titrated on HEL cell monolayers. CML1 was purified through a 36% sucrose cushion. Briefly, HEL cells were infected at a multiplicity of infection (MOI) of 0.01 for 2 hr in MEM 2% FCS, and then replaced in fresh MEM 2%. At day 2 post-infection, the supernatant was removed, cells were scraped in phosphate buffer saline (PBS) and frozen at −80°C. Infected cells were homogenized on ice in a glass dounce homogenizer (type A pestel) with 15 strokes. Cell debris were removed by centrifugation at 1200 rpm, 4°C, and the supernatant was layered on a cold 36% sucrose cushion. Following ultracentrifugation (13,500 rpm, 90 min, 4°C), the viral pellet was resuspended in 1 mM Tris-HCl pH 9.5 and crude virus stocks were stored at −80°C.

### Mice

Female athymic nude (*nu/nu*) NMRI mice were purchased from Elevage-Janvier (Le-Genest-St-Isle, France). All experimental and animal experimentations were completed at biosafety level 2. Animals were 13 to 15 g (4 to 5 week-old) the day of infection, and groups were defined as uninfected, CML1-infected, HPMPC-treated or HPMPDAP-treated. Depending on the experiment, 5 to 20 animals per group were used.

### Ethics Statement

All animal work was approved by the Katholieke Universiteit Leuven Ethics Committee for Animal Care and Use (Permit number: P044-2010). All animal guidelines and policies were in accordance with the Belgian Royal Decree of 14 November 1993 concerning the protection of laboratory animals and the European Directive 86-609-EEC for the protection of vertebrate animals used for experimental and other scientific purposes. Infections were performed under anesthesia using ketamine/xylazine in saline and, when required, euthanasia was done by administration of pentobarbital sodium.

### Animal infections

For i.n. infections, mice were anesthetized using a cocktail of ketamine/xylazine in saline, and inoculated with 10 µl of PBS containing 2.0×10^6^ PFU CML1 (5 µl per nostril) or mock-inoculated with 10 µl of PBS. For i.c. infections, mice received a light anesthesia (ketamine/xylazine in saline) and were immobilized manually by one person. Scarifications were made, as described elsewhere [55], by applying a 10 µl drop of 2.0×10^6^ PFU CML1 at the lumbosacral region.

### Animal's monitoring and scoring

In all experiments, mice were monitored for body weights, morbidity and mortality for 45 (i.n. model) or 75 days (i.c. model). In the i.c. model of infection, the development and propagation of the lesions were referred to a disease index scored on a 0 to 5 scale as follow: 0, absence of lesions; 1, appearance of tiny lesions; 2, visible lesions; 3, enlargement of lesion's size; 4, increase of lesion's severity (bleeding); 5, spreading of the lesions to the tail and/or the legs. Also, all deaths reported were due to sacrifices based on humane end-points in order to minimize pain and distress. For the i.n. model, animals were euthanized because of (i) failure in gaining weight as compared with uninfected controls (i.e., there was more than 35% difference in body weight when compared to uninfected controls) and (ii) severe sickness associated with development of lesions and/or edemas. Animals responding to these criteria were also prostrated. In the i.c. model, animals were euthanized based on severe disease development (high average disease index) with or without differences in body weight when compared to uninfected controls; animals met these criteria around day 75 post-infection.

### Antiviral compounds and treatments

(S)-HPMPC was provided by Gilead Sciences (Foster City, CA, US) and (S)-HPMPDAP was synthesized by Marcela Krečmerová at the Institute of Organic Chemistry and Biochemistry, Academy of Sciences of the Czech Republic, Prague. Animals infected via the i.c. route received cream-based treatments consisting of a 1% (S)-HPMPC- or (S)-HPMPDAP in cetomacrogol ointment. Treatments were applied by light finger-massages on the scarified zone once per day for 5 days, starting the day of scarification. For systemic administrations, working solutions were prepared in PBS at a concentration of 4 mg/ml. Each compound was administrated intraperitoneally at a dose of 50 mg/kg once per day for 3 days starting the day of infection.

### Tissue collection

To determine the extent of viral replication, 4 mice per group were euthanized at days 25 and 45 post-i.n. infection, and at days 30 and 75 post-i.c. infection. Heart punction was performed to collect blood. Serum was isolated following clotting of the blood at +4°C, centrifugation and supernatant was collected and stored at −20°C. Swabs of the skin of the lumbosacral region (i.c.), of the tail (i.c. and i.n.), and of the legs (i.n.) were collected in 500 µl PBS containing 2% FCS (PBS 2%) and stored at −20°C. Lymph nodes, spleen, liver, lungs, back skin, tail and leg were dissected aseptically. Lymph nodes and spleen were processed separately, and the isolated cells were used for flow cytometry and viral DNA quantification. Liver, lungs and back skin were collected in PBS 2%, weighed, frozen at −20°C, and homogenized prior DNA extraction. All DNA extractions were performed by using QIAamp^®^ DNA Mini and blood Mini kits (Qiagen, Venlo, Netherlands) following manufacturer's instructions, and samples were then subjected to quantitative real-time PCR (qPCR).

### Seroneutralization

Plaque neutralization assays were performed in 96-well microplates. Each serum was tested individually. Sera were diluted 1 10 in MEM 2%, inactivated for 30 min at 56°C and two-fold dilutions were done to reach 1 640. CML1, diluted in MEM 2% to a concentration of 100 TCID_50_ in 100 µl, was added to each well containing the sera to test and to the virus-control wells. The microplates were incubated for 2 h at 37°C. HEL cells, resuspended in MEM 2%, were then added at a concentration of 2.8×10^4^ cells per 80 µl per well. Microplates were incubated for 3 days at 37°C and examined for plaque formation. The neutralization titer was calculated using the Spearman-Karber formula which is the negative logarithm of the highest dilution of serum that caused a 50% reduction in the number of wells exhibiting CPE. The assays included virus control, cell control, negative controls of uninfected animals and controls to evaluate potential toxicity of the serum alone.

### Viral DNA detection

qPCR targeting the *CMP48L* gene (homolog of the *F13L* gene of VACV strain Copenhagen) was used to quantify viral DNA. qPCR was done on 5 µl of total DNA extracted in a 20 µl reaction volume using the TaqMan^®^ Fast Universal PCR master mix, forward primer [5′-CAA CTC CAT TAT AGA AGC CAT T-3′], reverse primer [5′-CGT VGT TCT TAT CCC AAT TAC CA-3′], and an MGB probe [6-FAM-ATA GAG GAG TTA AGA TCA GAC TT-MGB] (Applied Biosystems, Halle, Belgium and Warrington, UK). Reactions were processed by using the SDS7500Fast apparatus. Plasmid DNA containing the *CMP48L* gene was used to obtain the standard curve and to determine the DNA copy numbers per sample. The limit of detection per 5 µl sample was 10 DNA copies.

### Histopathology

One mouse of each group, i.e., group uninfected or group CML1, was euthanized and various tissue samples, such as skin, tail, leg, lungs, lymph nodes, were collected for histological examinations. Samples were fixed for one day in formol 6% and then placed in PBS. Samples were embedded in paraffin and 5 micrometer sections were stained with hematoxylin-eosin.

### Preparation of cell suspensions

Spleen and pool of draining lymph nodes (DLNs; inguinal, axillary, mesenteric and lumbar lymph nodes) were isolated from 4 individual mice in each group and processed separately. Organs were disrupted in cold PBS 2%, and filtered through a 70 µm nylon cell strainer. For all procedures, cell suspensions were kept on ice and washings were done with cold PBS 2%. Cell suspensions from the spleen were washed once, treated with ammonium chloride and washed again. Lymph node cells were only washed once. Cells were then counted before antibody (Ab) staining.

### Flow cytometry

The following conjugated Abs, purchased from eBioscience (San Diego, CA, USA), were used: anti-CD19-FITC (clone 1D3), anti-Ly-6G (Gr1)-FITC (clone RB6-8C5), anti-CD11c-FITC (clone N418), anti-CD45R (B220)-PE (clone RA3-6B2), anti-F4/80-PE (clone BM8), anti-CD11b-APC (clone M1/70), anti-pan-NK cells (CD49b)-APC (clone DX5). Cell suspensions obtained from the spleen and lymph nodes (10^6^ cells/tube) were blocked with a 1/3 dilution of FcR Blocking Reagent (Miltenyi Biotec, Bergisch Gladbach, Germany) for 20 min at 4°C, and then stained in PBS 2% for 20 min at 4°C with the Ab of interest. Cells were washed once with cold PBS 2% between each Ab staining, and washed twice before fixation with a 3% formaldehyde solution. Fluorescence was acquired on a total of 2×10^4^ cells (spleen) or 1.5×10^4^ cells (lymph nodes) with a FACSCalibur flow cytometer (BD Biosciences, San Jose, CA, USA) and the data were analyzed with the Cellquest software (BD Biosciences).

### Cytokine assays

The sera of each group of mice were used for cytokine detection. IL-1β, IL-2, IL-4, IL-5, IL-6, IL-10, IL-13, IL-17, IL-18, GM-CSF, IFN-γ, TNF-α, M-CSF were measured using the kit Bio-Plex Pro™ Mouse cytokine multiplex Assay (Biorad, Nazareth Eke, Belgium), according to manufacturer's instructions. IL-6 immunoassays were performed using the kit Quantitine® (R&D Systems, Minneapolis, US), according to manufacturer's instructions. Only cytokine levels over 10 pg/ml were considered for statistical analyses.

### Statistical analyses

All statistical analyses were done with GraphPad Prism 5 software (GraphPad Software Inc., La Jolla, CA, USA). Viral loads and FACS results were analyzed with Mann Whitney test and unpaired Student *t* test, respectively, and survival curves with Log-rank (Mantel-Cox) test. Statistic significance was defined as follow: *p*<0.001 or ***, extremely significant; *p*<0.01 or **, very significant; *p*<0.05 or *, significant; and *p*>0.05 or ns, not significant.

## References

[1] Afonso CL, Tulman ER, Lu Z, Zsak L, Sandybaev NT (2002). The genome of camelpox virus.. Virology.

[2] Gubser C, Smith GL (2002). The sequence of camelpox virus shows it is most closely related to variola virus, the cause of smallpox.. J Gen Virol.

[3] Wernery U, Zachariah R (1999). Experimental camelpox infection in vaccinated and unvaccinated dromedaries.. Zentralbl Veterinarmed B.

[4] Al Zi'abi O, Nishikawa H, Meyer H (2007). The first outbreak of camelpox in Syria.. J Vet Med Sci.

[5] Wernery U, Kaaden OR (2002). Infectious diseases in camelids..

[6] Davies FG, Mungai JN, Shaw T (1975). Characteristics of a Kenyan camelpox virus.. J Hyg (Lond).

[7] Jezek Z, Kriz B, Rothbauer V (1983). Camelpox and its risk to the human population.. J Hyg Epidemiol Microbiol Immunol.

[8] Kriz B (1982). A study of camelpox in Somalia.. J Comp Pathol.

[9] Bera BC, Shanmugasundaram K, Barua S, Venkatesan G, Virmani N (2011). Zoonotic cases of camelpox infection in India..

[10] von Bomhard W, Mauldin EA, Breuer W, Pfleghaar S, Nitsche A (2010). Localized cowpox infection in a 5-month-old Rottweiler.. Vet Dermatol.

[11] Ninove L, Domart Y, Vervel C, Voinot C, Salez N (2009). Cowpox virus transmission from pet rats to humans, France.. Emerg Infect Dis.

[12] Kurth A, Straube M, Kuczka A, Dunsche AJ, Meyer H (2009). Cowpox virus outbreak in banded mongooses (Mungos mungo) and jaguarundis (Herpailurus yagouaroundi) with a time-delayed infection to humans.. PLoS One.

[13] Campe H, Zimmermann P, Glos K, Bayer M, Bergemann H (2009). Cowpox virus transmission from pet rats to humans, Germany.. Emerg Infect Dis.

[14] Glatz M, Richter S, Ginter-Hanselmayer G, Aberer W, Mullegger RR (2010). Human cowpox in a veterinary student.. Lancet Infect Dis.

[15] Bhanuprakash V, Venkatesan G, Balamurugan V, Hosamani M, Yogisharadhya R (2009). Zoonotic Infections of Buffalopox in India.. Zoonoses Public Health.

[16] Abrahao JS, Silva-Fernandes AT, Lima LS, Campos RK, Guedes MI (2010). Vaccinia virus infection in monkeys, Brazilian Amazon.. Emerg Infect Dis.

[17] Silva DC, Moreira-Silva EA, Gomes JA, Fonseca FG, Correa-Oliveira R (2010). Clinical signs, diagnosis, and case reports of Vaccinia virus infections.. Braz J Infect Dis.

[18] Rimoin AW, Mulembakani PM, Johnston SC, Lloyd Smith JO, Kisalu NK (2010). Major increase in human monkeypox incidence 30 years after smallpox vaccination campaigns cease in the Democratic Republic of Congo.. Proc Natl Acad Sci U S A.

[19] Abu Elzein EM, Gameel AA, Ramadan RO, Housawi FM (1999). An eruptive moderate form of camelpox infection in dromedary camels (Camelus dromedarius) in Saudi Arabia.. Rev Sci Tech.

[20] Kinne J, Cooper JE, Wernery U (1998). Pathological studies on camelpox lesions of the respiratory system in the United Arab Emirates (UAE).. J Comp Pathol.

[21] Otterbein CK, Meyer H, Renner-Muller IC, Munz E (1996). In vivo and in vitro characterization of two camelpoxvirus isolates with decreased virulence.. Rev Elev Med Vet Pays Trop.

[22] Bhanuprakash V, Balamurugan V, Hosamani M, Venkatesan G, Chauhan B (2010). Isolation and characterization of Indian isolates of camel pox virus.. Trop Anim Health Prod.

[23] Hafez SM, al Sukayran A, dela CD, Mazloum KS, al Bokmy AM (1992). Development of a live cell culture camelpox vaccine.. Vaccine.

[24] Pfeffer M, Meyer H, Wernery U, Kaaden OR (1996). Comparison of camelpox viruses isolated in Dubai.. Vet Microbiol.

[25] Duraffour S, Snoeck R, de Vos R, van Den Oord JJ, Crance JM (2007). Activity of the anti-orthopoxvirus compound ST-246 against vaccinia, cowpox and camelpox viruses in cell monolayers and organotypic raft cultures.. Antivir Ther.

[26] Duraffour S, Vigne S, Vermeire K, Garcel A, Vanstreels E (2008). Specific targeting of the F13L protein by ST-246 affects orthopoxvirus production differently.. Antivir Ther.

[27] Yang G, Pevear DC, Davies MH, Collett MS, Bailey T (2005). An orally bioavailable antipoxvirus compound (ST-246) inhibits extracellular virus formation and protects mice from lethal orthopoxvirus Challenge.. J Virol.

[28] Duraffour S, Snoeck R, Krecmerova M, Van den OJ, de Vos R (2007). Activities of several classes of acyclic nucleoside phosphonates against camelpox virus replication in different cell culture models.. Antimicrob Agents Chemother.

[29] Duraffour S, Andrei G, Snoeck R (2010). Tecovirimat, a p37 envelope protein inhibitor for the treatment of smallpox infection.. IDrugs.

[30] Hostetler KY (2009). Alkoxyalkyl prodrugs of acyclic nucleoside phosphonates enhance oral antiviral activity and reduce toxicity: current state of the art.. Antiviral Res.

[31] Centers for Disease Control and Prevention (CDC) (2007). Household transmission of vaccinia virus from contact with a military smallpox vaccinee--Illinois and Indiana, 2007.. MMWR Morb Mortal Wkly Rep.

[32] Centers for Disease Control and Prevention (CDC) (2009). Progressive vaccinia in a military smallpox vaccinee - United States, 2009.. MMWR Morb Mortal Wkly Rep.

[33] Centers for Disease Control and Prevention (CDC) (2009). Human vaccinia infection after contact with a raccoon rabies vaccine bait - Pennsylvania, 2009.. MMWR Morb Mortal Wkly Rep.

[34] Bristol N (2007). Adverse event related to smallpox vaccination.. Disaster Med Public Health Prep.

[35] Kaiser J (2007). Smallpox vaccine. A tame virus runs amok.. Science.

[36] Marris E (2007). Dramatic rescue relieves rare case of smallpox infection.. Nat Med.

[37] Vora S, Damon I, Fulginiti V, Weber SG, Kahana M (2008). Severe eczema vaccinatum in a household contact of a smallpox vaccinee.. Clin Infect Dis.

[38] Baxby D (1972). Smallpox-like viruses from camels in Iran.. Lancet.

[39] Falluji MM, Tantawi HH, Shony MO (1979). Isolation, identification and characterization of camelpox virus in Iraq.. J Hyg (Lond).

[40] Ramyar H, Hessami M (1972). Isolation, cultivation and characterization of camel pox virus.. Zentralbl Veterinarmed B.

[41] Sehgal CL, Ray SN (1980). Studies on camelpox virus.. J Commun Dis.

[42] Tantawi HH, El Dahaby H, Fahmy LS (1978). Comparative studies on poxvirus strains isolated from camels.. Acta Virol.

[43] Tscharke DC, Reading PC, Smith GL (2002). Dermal infection with vaccinia virus reveals roles for virus proteins not seen using other inoculation routes.. J Gen Virol.

[44] Osorio JE, Iams KP, Meteyer CU, Rocke TE (2009). Comparison of monkeypox viruses pathogenesis in mice by in vivo imaging.. PLoS One.

[45] Stabenow J, Buller RM, Schriewer J, West C, Sagartz JE (2010). A mouse model of lethal infection for evaluating prophylactics and therapeutics against Monkeypox virus.. J Virol.

[46] Americo JL, Moss B, Earl PL (2010). Identification of wild-derived inbred mouse strains highly susceptible to monkeypox virus infection for use as small animal models.. J Virol.

[47] Damon I, Knipe DM, Howley PM (2007). Poxviruses.. Fields Virology.

[48] Chapman JL, Nichols DK, Martinez MJ, Raymond JW (2010). Animal models of orthopoxvirus infection.. Vet Pathol.

[49] Ramshaw IA, Ramsay AJ, Karupiah G, Rolph MS, Mahalingam S (1997). Cytokines and immunity to viral infections.. Immunol Rev.

[50] Smee DF, Gowen BB, Wandersee MK, Wong MH, Skirpstunas RT (2008). Differential pathogenesis of cowpox virus intranasal infections in mice induced by low and high inoculum volumes and effects of cidofovir treatment.. Int J Antimicrob Agents.

[51] Liu G, Zhai QZ, Schaffner DJ, Wu AG, Yohannes A (2004). Prevention of lethal respiratory vaccinia infections in mice with interferon-alpha and interferon-gamma.. Fems Immunology and Medical Microbiology.

[52] Quenelle DC, Collins DJ, Kern ER (2004). Cutaneous infections of mice with vaccinia or cowpox viruses and efficacy of cidofovir.. Antiviral Res.

[53] Smee DF, Bailey KW, Wong MH, Wandersee MK, Sidwell RW (2004). Topical cidofovir is more effective than is parenteral therapy for treatment of progressive vaccinia in immunocompromised mice.. Journal of Infectious Diseases.

[54] Grosenbach DW, Berhanu A, King DS, Mosier S, Jones KF (2010). Efficacy of ST-246 versus lethal poxvirus challenge in immunodeficient mice.. Proc Natl Acad Sci U S A.

[55] Neyts J, Leyssen P, Verbeken E, De Clercq E (2004). Efficacy of cidofovir in a murine model of disseminated progressive vaccinia.. Antimicrob Agents Chemother.

[56] Thin G (1882). The Histology of Molluscum Contagiosum.. J Anat Physiol.

[57] Bayer-Garner IB (2005). Monkeypox virus: histologic, immunohistochemical and electron-microscopic findings.. J Cutan Pathol.

[58] Schoepp RJ, Morin MD, Martinez MJ, Kulesh DA, Hensley L (2004). Detection and identification of Variola virus in fixed human tissue after prolonged archival storage.. Lab Invest.

[59] Karupiah G, Buller RM, Van Rooijen N, Duarte CJ, Chen J (1996). Different roles for CD4+ and CD8+ T lymphocytes and macrophage subsets in the control of a generalized virus infection.. J Virol.

[60] Chaudhri G, Panchanathan V, Buller RM, van den Eertwegh AJ, Claassen E (2004). Polarized type 1 cytokine response and cell-mediated immunity determine genetic resistance to mousepox.. Proc Natl Acad Sci U S A.

[61] Buller RM, Palumbo GJ (1991). Poxvirus pathogenesis.. Microbiol Rev.

[62] Reading PC, Smith GL (2003). A kinetic analysis of immune mediators in the lungs of mice infected with vaccinia virus and comparison with intradermal infection.. J Gen Virol.

[63] O'Gorman WE, Sampath P, Simonds EF, Sikorski R, O'Malley M (2010). Alternate mechanisms of initial pattern recognition drive differential immune responses to related poxviruses.. Cell Host Microbe.

[64] Abadie V, Bonduelle O, Duffy D, Parizot C, Verrier B (2009). Original encounter with antigen determines antigen-presenting cell imprinting of the quality of the immune response in mice.. PLoS One.

[65] Tscharke DC, Woo WP, Sakala IG, Sidney J, Sette A (2006). Poxvirus CD8+ T-cell determinants and cross-reactivity in BALB/c mice.. J Virol.

[66] Knorr CW, Allen SD, Torres AR, Smee DF (2006). Effects of cidofovir treatment on cytokine induction in murine models of cowpox and vaccinia virus infection.. Antiviral Res.

[67] Nakanishi K, Yoshimoto T, Tsutsui H, Okamura H (2001). Interleukin-18 is a unique cytokine that stimulates both Th1 and Th2 responses depending on its cytokine milieu.. Cytokine Growth Factor Rev.

[68] Reading PC, Smith GL (2003). Vaccinia virus interleukin-18-binding protein promotes virulence by reducing gamma interferon production and natural killer and T-cell activity.. J Virol.

[69] Alcami A, Smith GL (1995). Vaccinia, cowpox, and camelpox viruses encode soluble gamma interferon receptors with novel broad species specificity.. J Virol.

[70] Najarro P, Traktman P, Lewis JA (2001). Vaccinia virus blocks gamma interferon signal transduction: viral VH1 phosphatase reverses Stat1 activation.. J Virol.

[71] Smee DF (2008). Progress in the discovery of compounds inhibiting orthopoxviruses in animal models.. Antivir Chem Chemother.

[72] Gammon DB, Snoeck R, Fiten P, Krecmerova M, Holy A (2008). Mechanism of antiviral drug resistance of vaccinia virus: identification of residues in the viral DNA polymerase conferring differential resistance to antipoxvirus drugs.. J Virol.

[73] Gubser C, Bergamaschi D, Hollinshead M, Lu X, van Kuppeveld FJ (2007). A new inhibitor of apoptosis from vaccinia virus and eukaryotes.. PLoS Pathog.

[74] Gubser C, Goodbody R, Ecker A, Brady G, O'Neill LA (2007). Camelpox virus encodes a schlafen-like protein that affects orthopoxvirus virulence.. J Gen Virol.

